# Granulocytic Sarcoma of Parotid Gland in a 4-Year-Old Child with Subleukemic AML: A Diagnostic Challenge!

**DOI:** 10.1155/2013/321289

**Published:** 2013-09-04

**Authors:** Yashwant Ingale, Tushar Patil, Priyanka Chaudhari, Samapika Routray, Manoj Agrawal

**Affiliations:** ^1^Department of Dentistry, Yashwantrao Chavan Memorial Hospital, Pimpri, Pune 411018, India; ^2^Department of Pathology, Yashwantrao Chavan Memorial Hospital, Pimpri, Pune 411018, India; ^3^Department of Oral Pathology & Microbiology, Institute of Dental Sciences, SOA University, Bhubaneswar, Odisha 751003, India; ^4^Oral & Maxillofacial Surgeon, Yashwantrao Chavan Memorial Hospital, Pimpri, Pune 411018, India

## Abstract

A 4-year-old male child presented to our outpatient department with large swelling in the parotid region. Routine investigations were all within normal limits, and evaluation of complete blood count was normal except for anaemia. Excisional biopsy as a therapeutic diagnosis was done. Microscopic examination showed monomorphic population of discohesive, hyperchromatic small round cells having high N : C ratio, coarse chromatin, conspicuous nucleoli, and sometimes angulated nuclei lying in sheets. Immunohistochemistry was done to rule out possible differential diagnosis. Fine needle aspiration from the swelling showed predominant population of blast cells. Myeloperoxidase and PBO were strongly positive, and diagnosis of granulocytic sarcoma was confirmed.

## 1. Introduction:

Granulocytic sarcoma (GS) also earlier referred to as chloroma or myeloblastoma is an extra medullary tumour composed of granulocytic precursor cells. The term granulocytic sarcoma was coined by Rappaport in 1966 [1]. More recently, the term extramedullary myeloid tumour has evolved.

Granulocytic sarcoma occurs in various settings [2] as follows:

- in association with acute myeloid leukaemia,
- in nonleukemic patients with normal peripheral blood and bone marrow findings, but who in due course of time develop acute myeloid leukaemia,
- in myelodysplastic syndrome (MDS) with leukemic transformation,
- in association with myeloproliferative disorders, where it heralds the onset of blastic transformation.

## 2. Case Report

**Figure 1:**
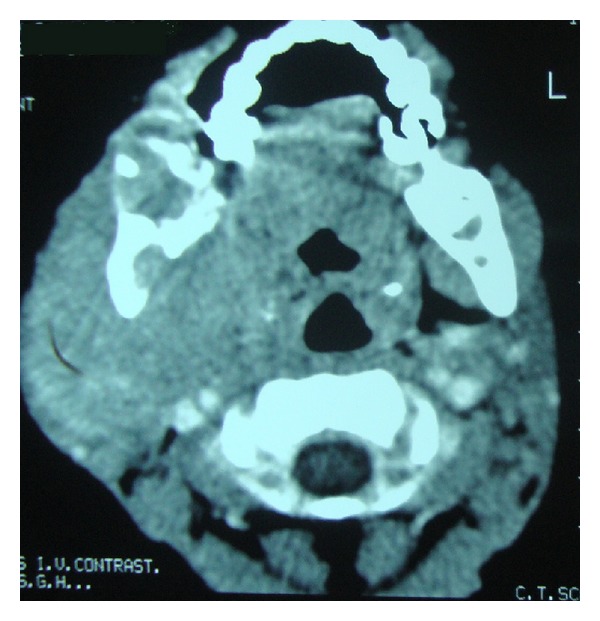
Computed tomography showing the extensions of the lesion.

**Figure 2:**
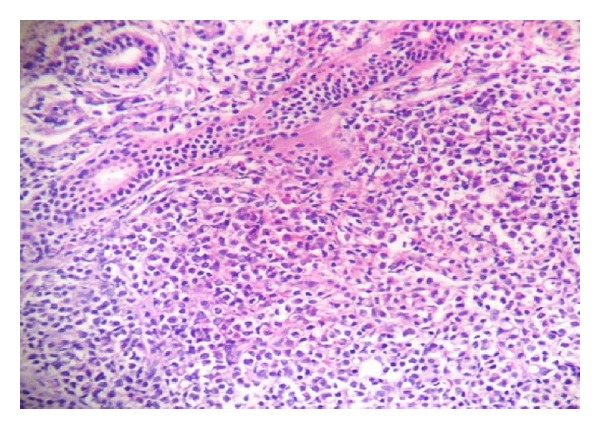
Parotid ducts with infiltrating tumour under low-power view.

**Figure 3:**
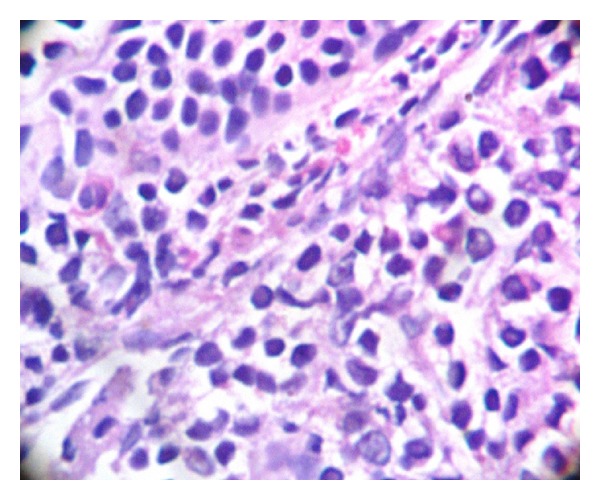
High-power view showing blast (tumour) cells which are round to oval with high nuclear cytoplasmic ratio and increased eosinophils.

**Figure 4:**
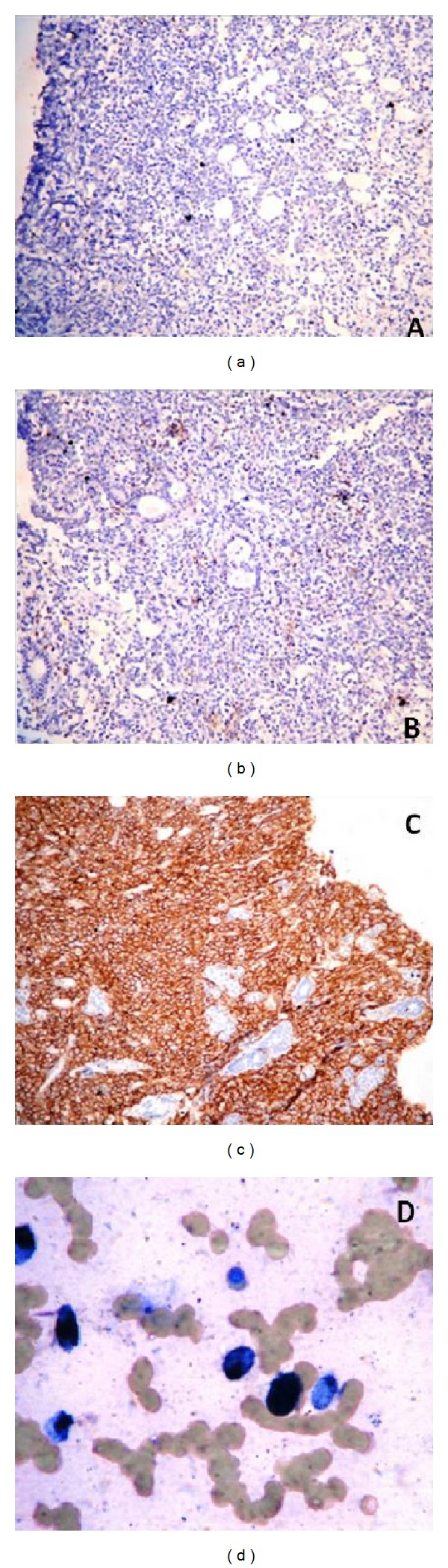
Immunohistochemistry showed (a) S100 negative which ruled out LCH. (b) CD3 negative ruled out T-cell lymphoma. (c) CD43 positive in all neoplastic cells. (d) Myeloblasts are demonstrated in PBS by myeloperoxidase stain.

A four-year-old febrile healthy male child presented with a lump in the right parotid region. On examination, the swelling was about 6 × 5 cm in size, nontender, irregular, and firm to hard in consistency with obvious proptosis. Swelling extended superiorly up to zygomatic region, inferiorly up to mandible, anteriorly up to maxillary region, and posteriorly up to retromandibular space. Radiological examination showed a large solid ill-defined lesion along the ramus of the mandible extending into masseteric space and until skull base of the right side. Involvement of nasopharynx, posterior ethmoid, and sphenoid sinus with sinus wall remodelling was suggestive of neoplastic mass (Figure 1). Clinical differential diagnosis of rhabdomyosarcoma, lymphoma, and primitive neuroectodermal tumour was suggested. Routine investigations were all within normal limits, and evaluation of complete blood count was normal except for anaemia. On microscopic examination of the processed biopsy, tissue sections showed mainly tumour tissue infiltrating into normal parotid gland (Figure 2). Tumour cells seen were mostly round to oval with high N/C ratio and hyperchromatic with irregular nuclear membrane and at some places with angulated nuclei also (Figure 3). Increased number of eosonophilic precursors were also noted along with few neutrophils and lymphoid cells. No evidence of acinous formation or lymphoglandular bodies was found which helped in ruling out carcinoma of parotid gland or lymphoma. Histopathological diagnosis of Langerhans cell histiocytosis (LCH) versus granulocytic sarcoma was considered. To confirm the histologic diagnosis and to rule out clinical diagnosis, immunohistochemistry was performed, and a panel of markers were used as described in Table 1 and Figure 4. Peripheral blood smear showed increased number of blast cells, morphologically mostly monoblast and myeloblast in myeloperoxidase staining (MPO). The bone marrow smear taken showed increased number of blast cells (more than 20%) which were myeloperoxidase (MPO) and lysozyme positive. Serum LDH and serum uric acid levels were also found to be elevated. Final diagnosis of granulocytic sarcoma of parotid gland in the case of subleukemic AML was given on the basis of history, clinical, haematological investigation, and histological studies supported by immunohistochemistry data.

## 3. Discussion

Granulocytic sarcoma (GS) is characterized by localized infiltration of immature granulocytes in an extramedullary site or soft tissue, and it was first described in the early 1800s as a tumour on the retroorbital site with a green-colour appearance, which caused proptosis [3]. The most frequent chromosomal abnormality associated with certain myeloid sarcomas has been observed to be t(8;21)(q22;q22), an abnormality that it shares with some AMLs [4]. GS can also occur as a precursor to AML rarely without any evidence of a haematological disorder [5]. In our MEDLINE search, only 4 cases of GS were shown involving parotid gland till date. As our case report was a rare instance, we further confirmed the diagnosis based on immunohistochemistry data as it aids in distinguishing myeloid sarcoma from malignant lymphoma; however, the coexpression of some T-cell markers and staining with TdT and CD 34 can cause difficulties in interpretation. The best immunohistochemical stains used included MPO and lysozyme. MPO immunostain is positive in most myeloblastic variants (as well as in some cells myelomonocytic variants), while lysozyme is frequently expressed in monoblastic variants. In patients with AML, the progression of myeloid sarcoma has the same prognosis as the underlying leukaemia. Patients with an AML associated with a t(8;21) and presenting myeloid sarcoma have a low rate of complete remission, and overall survival is poor. This appears to be in contrast to the better prognosis generally seen in AML with t(8;21). In patients with chronic myeloproliferative disorders (CMPD) and myelodysplastic disorders (MDS), myeloid sarcoma defines a blastic transformation often associated with a short survival.

## 4. Conclusion

 The GS is a rare neoplasm in this early age group especially in the head and neck region, and it is frequently confused with other tumours. The diagnosis of the GS is always challenging for both the physician and the pathologist, mainly when it is not associated with the myeloid leukaemia, when it is necessary to differentiate it from other malignant tumours, such as the lymphomas. The careful morphological analysis and the staining by immunohistochemistry are vital for establishing the diagnosis. Even though this lesion is rarely found in the oral cavity, its occurrence may never be ignored.

## Figures and Tables

**Table 1:** Immunohistochemical markers used to rule out the differential diagnosis in our case.

Marker	Result	Diagnosis excluded
S100	Negative	LCH
CD3	Negative	T-cell lymphoma
CD79 a	Negative	B-cell lymphoma
Lysosome	Positive	
CD43	Positive	

## References

[1] Rappaport H (1966). *Tumors of Hemopoieticsystem: Atlas of Tumour Pathology. Section 3. Fascicle 8*.

[2] Neiman RS, Barcos M, Berard C (1981). Granulocytic sarcoma: a clinicopathologic study of 61 biopsied cases. *Cancer*.

[3] Eshghabadi M, Shojania AM, Carr I (1986). Isolated granulocytic sarcoma: report of a case and review of the literature. *Journal of Clinical Oncology*.

[4] Tanigawa M, Tsuda Y, Amemiya T, Yamada K, Nakayama M, Tsuji Y (1998). Orbital tumor in acute myeloid leukemia associated with karyotype 46,XX,t(8;21)(q22;q22): a case report. *Ophthalmologica*.

[5] Srinivasan B, Ethunandan M, Anand R, Hussein K, Ilankovan V (2008). Granulocytic sarcoma of the lips: report of an unusual case. *Oral Surgery, Oral Medicine, Oral Pathology, Oral Radiology and Endodontology*.

